# Metabolomics-Based Analysis Reveals Flavonoid-Mediated Defence in Alfalfa (*Medicago sativa* ‘Gannong No. 5’) Against Pea Aphid (*Acyrthosiphon pisum*) Infestation

**DOI:** 10.3390/insects17070679

**Published:** 2026-06-30

**Authors:** Huan Liu, Zhan-Zi Yu, Qin-Zhe Sun, Lei Liu, Xing Xiang, Li-Wen Song, Sen-Shan Wang, Wan-Bin Chen

**Affiliations:** 1 Biocontrol Engineering Laboratory of Crop Diseases and Pests of Gansu Province, College of Plant Protection, Gansu Agricultural University, Lanzhou 730070, China; 14059@gsau.edu.cn (H.L.);; 2 Institute of Plant Protection, Gansu Academy of Agricultural Sciences, Lanzhou 730070, China

**Keywords:** *Acyrthosiphon pisum*, Gannong No. 5, metabolomics analysis, flavonoids, sakuranetin, chrysin, antibiosis

## Abstract

The pea aphid (*Acyrthosiphon pisum*) is a destructive phloem-feeding pest of alfalfa. Its overreliance on chemical insecticides necessitates the development of sustainable, plant-based resistance strategies. Plant flavonoids are important in induced defense, but their dynamic responses to aphid feeding and subsequent insecticidal effects remain unclear. Using the highly resistant alfalfa variety ‘Gannong No. 5’ (GN5), this study integrated behavioral assays, performance bioassays, untargeted and targeted metabolomics, and exogenous flavonoid feeding assays to investigate how pea aphid infestation alters flavonoid metabolism and thereby affects aphid performance. Bioassays confirmed strong antibiosis of GN5 against *A. pisum*. Behavioral choice assays showed that, at 2 h post-release, aphids significantly avoided plants pre-infested for 12 h and 48 h, but not those pre-infested for 24 h; by 8 h post-release, they significantly avoided all pre-infested plants regardless of infestation duration. Prolonged pre-infestation (48 h) also reduced average fecundity per female. Non-targeted metabolomics revealed substantial metabolic reprogramming after 48 h of aphid feeding, with most flavonoids and isoflavonoids significantly upregulated. Targeted metabolomics identified 28 flavonoids, among which only sakuranetin and chrysin were significantly upregulated after 48 h, indicating their specific induction. Finally, performance bioassays confirmed insecticidal effects in a concentration-dependent manner: sakuranetin at 0.1 μg/μL reduced reproduction, and at 10.0 μg/μL reduced survival; chrysin at 0.1 μg/μL reduced both survival and reproduction. Collectively, these results demonstrate that pea aphid feeding triggers the induction of specific defensive flavonoids in GN5, which may contribute to antibiosis. This study provides a theoretical basis for exploring flavonoid-based approaches in sustainable aphid management.

## Simple Summary

The pea aphid (*Acyrthosiphon pisum*) is a major pest of alfalfa, an important forage crop. Farmers often rely heavily on chemical insecticides for control, but overuse harms the environment and leads to resistance. Therefore, eco-friendly, plant-based strategies are urgently needed. In this study, the response of a highly aphid-resistant alfalfa variety, ‘Gannong No. 5’ (GN5), to pea aphid feeding was examined. Its infestation induced the accumulation of specific flavonoids, including sakuranetin and chrysin, in GN5 leaves. In separate feeding assays using detached leaves, the presence of these flavonoids correlated with reduced aphid survival and reproduction in a concentration-dependent manner. These findings suggest that sakuranetin and chrysin may contribute to GN5’s resistance, and thus could serve as potential leads for plant-derived aphid management agents.

## 1. Introduction

The pea aphid (*Acyrthosiphon pisum*) is a small but highly destructive pest that infests important forage legumes, including alfalfa (*Medicago sativa*), faba bean (*Vicia faba*), and lentil (*Lens culinaris*) [1]. Using its piercing–sucking mouthparts to feed on phloem sap, *A. pisum* causes nutrient loss, growth retardation, and plant malformation. Additionally, the aphid acts as a vector for more than 20 plant viruses, inducing symptoms including stunting, mosaics, and mottling, thereby posing a serious threat to global leguminous crop production [2,3,4]. To mitigate these direct and indirect damages, chemical control has long been the primary strategy for managing pea aphid populations [1]. However, frequent and intensive application of chemical insecticides has led to the “3R” problems (resistance, resurgence, and residue) and impairs soil microbial activity, severely constraining sustainable agricultural development. Moreover, pesticide residues can enter livestock and poultry via contaminated forage, threatening human health through the food chain [5]. Consequently, the overreliance on chemical insecticides highlights the urgent need for sustainable and eco-friendly alternatives to manage pea aphids.

Host plant resistance plays a key role in pest management. When attacked by herbivorous insects, plants activate a sophisticated defense system [6]. Plant resistance to insects can be classified into three categories based on mechanism—antibiosis, antixenosis, and tolerance—or into two categories based on temporal expression—constitutive and induced resistance. Antibiosis is a critical component of plant defense, involving secondary metabolites, nutrients, and other substances that impair insect digestion [7]. To date, over 50,000 flavonoids have been identified in plants, including major classes such as flavanones, flavones, isoflavones, flavonols, anthocyanins, and flavan-3-ols [8,9]. Moreover, more than 280 flavonoids have been reported to possess insecticidal activity [10]. These compounds are often induced following herbivore attack and can exert antifeedant and repellent effects, or directly disrupt insect metabolism, thereby inhibiting oviposition, feeding, and development [11,12,13,14]. In rice, flavonoid content is positively correlated with resistance to the brown planthopper (*Nilaparvata lugens*), and exogenous application of flavonoids significantly enhances resistance in wild-type rice [15]. The flavone schaftoside binds to and inhibits its kinase NlCDK1 of *N. lugens*, impairing ovarian development, reducing fecundity, and decreasing survival [16,17]. Furthermore, tricin reduces larval survival and development, decreases adult fecundity and egg hatchability, and disrupts phloem feeding in *N. lugens* [18,19]. In tea plants, infestation by the tea geometrid (*Ectropis grisescens*) induces significant increases in (+)-catechin, epicatechin, and epigallocatechin. An artificial diet containing these three flavonoids markedly retards larval growth, confirming that catechins are important antibiotic compounds in tea plants against this pest [20]. In cotton, phlorizin and dihydroquercetin significantly reduce adult survival and fecundity of the cotton aphid (*Aphis gossypii*) [21]; naringenin, apigenin, and kaempferol hinder colonization on host plants, suppress feeding behavior, and delay population expansion [9]. Pinocembrin, quercetin, luteolin, and certain flavonoid derivatives exhibit insecticidal activity against the fall armyworm (*Spodoptera frugiperda*) [22,23].

Flavonoids, as characteristic metabolites of leguminous plants, play an important role in anti-herbivore defense. For example, the flavonoid-rich alfalfa variety (Radius) suppresses feeding and reproduction of the pea aphid [24]. Further investigation revealed that apigenin glycosides in alfalfa are negatively correlated with pea aphid population size and phloem sap ingestion, suggesting that these flavonoid glycosides may serve as important nutritional or defensive factors mediating alfalfa resistance to aphids [25]. When pea aphids were fed an artificial diet supplemented with naringenin—a flavonoid biosynthetic precursor—their fecundity and intrinsic rate of increase were significantly reduced [26]. In alfalfa, flavonoid accumulation is significantly induced by insect infestation. Studies have demonstrated that after infestation by pea aphids or thrips, the flavonoid and isoflavonoid biosynthesis pathways are markedly activated, with multiple flavonoids—naringenin, apigenin and luteolin—being upregulated [27]. In other leguminous crops, similar effects have been observed. Genistein inhibits the development of the redbanded stink bug (*Piezodorus guildinii*) in soybean [28]. Gels containing naringenin and genistein significantly inhibit feeding by the black bean aphid (*Aphis fabae*) [29]. Genistein has also been identified as a key compound mediating resistance in peanut to the western flower thrips (*Frankliniella occidentalis*) [30]. In addition, a synergistic effect may exist between flavonoids and other defensive substances such as saponins in alfalfa, as alfalfa lines with high saponin content also suppress the feeding behavior and reproduction of the pea aphid [31]. Nevertheless, studies on the potential insecticidal activity of flavonoids in alfalfa remain limited.

Alfalfa (*Medicago sativa*) is renowned for its strong nitrogen-fixing ability and high leaf crude protein content. Although alfalfa has been cultivated in China for over 2000 years, with a preserved planting area of 3.774 million hectares [32], domestic production meets less than half of the demand, with the rest relying on imports. This supply–demand gap poses a significant constraint on the development of China’s livestock industry. Because alfalfa is grown for direct animal consumption, chemical insecticides are discouraged in its pest management [33]. Fortunately, alfalfa is rich in saponins, flavonoids, polysaccharides, and coumarins, which exhibit anti-inflammatory, antibacterial, and insecticidal activities [24,34], making this plant an ideal source of biopesticides. Therefore, harnessing insecticidal secondary metabolites from alfalfa and developing transgenic aphid-resistant varieties represent promising approaches to reduce pesticide use in alfalfa cultivation.

‘Gannong No. 5’ (GN5) is the first alfalfa variety developed in China exhibiting both high resistance to aphids and tolerance to thrips damage. This variety was bred by the College of Grassland Science at Gansu Agricultural University and was officially approved by the National Forage Variety Registration Committee in April 2010 [35]. To investigate the metabolic response of GN5 to aphid infestation and identify candidate secondary metabolites with activity against the pea aphid, a series of laboratory bioassays were first conducted to evaluate aphid preference and performance on aphid-infested plants. Subsequently, both widely targeted and targeted metabolomic analyses were employed to characterize changes in flavonoid metabolites in alfalfa leaves following aphid infestation. Finally, candidate metabolites were screened for insecticidal activity against pea aphid via exogenous feeding assays. This study provides a theoretical basis for the development of novel, green, plant-derived insecticides for the sustainable management of *A. pisum*.

## 2. Materials and Methods

### 2.1. Insects and Plants

Seeds of alfalfa cultivar ‘Gannong No. 5’ (GN5) and ‘Hunter River’ (HR) were soaked in tap water and allowed to germinate at 25 ± 1 °C for two days. They were then sown in seedling cups (7.5 cm diameter, 9 cm height) filled with a substrate of soil: peat: vermiculite (3:2:1, *v*/*v*/*v*), with one plant per cup. The cups were placed in a climate-controlled chamber under the following conditions: temperature 25 ± 1 °C, relative humidity (RH) 70 ± 5%, and photoperiod 16 h light:8 h dark. Plants were grown for 50 days prior to the experiment.

Seeds of faba bean cultivar ‘Lincan No. 9’ were soaked in tap water for approximately three days and planted after germination in perforated plastic pots (13 cm diameter, 12 cm height), with six seeds per pot, covered with the same substrate. The pots were maintained in a climate chamber under the same conditions (25 ± 1 °C, 70 ± 5% RH, 16 h light:8 h dark photoperiod).

*Acyrthosiphon pisum* used in this study were collected from alfalfa fields in Lanzhou New District (Lanzhou, Gansu Province, China; 36°35′ N, 103°38′ E) in June 2025 and reared in a laboratory climate chamber on the susceptible alfalfa cultivar ‘Hunter River’ [35] under conditions of 20 ± 1 °C, 70 ± 5% RH, and a 16 h light:8 h dark photoperiod.

### 2.2. Alfalfa Plants Infested with A. pisum

Fifty-day-old alfalfa plants were placed in transparent plastic cylinders (1.3 cm diameter, 25 cm height), with the bottom end of each cylinder inserted into the soil. Fifty third-instar *A. pisum* were introduced into each cylinder to feed on the alfalfa leaves, and the top of the cylinder was sealed with absorbent cotton to prevent aphid escape. At 12, 24, and 48 h post-infestation (hpi), the nymphs were gently removed from the leaves with a soft brush, and the leaves that had been exposed to aphid feeding (designated as TGN5) were collected for subsequent biological assays. For metabolomic analysis, leaves were uniformly collected from the top to the bottom of the plant at 48 hpi using sterile scissors, transferred into 1.5 mL centrifuge tubes, immediately flash-frozen in liquid nitrogen, and subsequently stored at −80 °C until use. Alfalfa plants placed in cylinders without aphid infestation served as controls (designated as CGN5, collected at 0 hpi).

### 2.3. Acyrthosiphon pisum Preference to Conspecific-Infested Alfalfa Plants

To investigate the effect of prior feeding on subsequent host preference in pea aphids, we adopted the method of Liu et al. [36]. Lateral branches from alfalfa plants that had been pre-infested by pea aphids for 0 (uninfested), 12, 24, or 48 h were excised as described in Section 2.2. The cut ends were wrapped with moist absorbent cotton, and branches were placed at equal distances in a 15 cm Petri dish lined with moist filter paper. Each dish contained one pair of branches (e.g., Ap_12 h *vs*. Ap_0 h, Ap_24 h *vs*. Ap_0 h, and Ap_48 h *vs*. Ap_0 h). Twenty apterous adult aphids were introduced into the center of the dish. The number of aphids settling on alfalfa branches from each infestation time was recorded at 2 and 8 h after release. Aphids that were walking on the Petri dish or did not settle on either branch within the observation period were recorded as “non settled” and were excluded from the denominator when calculating preference percentages. The experiment was conducted in a climate-controlled chamber under the same conditions as those used for rearing (20 ± 1 °C, 70 ± 5% RH, and a 16 h light:8 h dark photoperiod). To avoid positional effects on host preference, the positions of the treatments and control branches were rotated between replicates. Six biological replicates were performed.

### 2.4. Acyrthosiphon pisum Performance on Conspecific-Infested Alfalfa Plants

To further evaluate the effects of pre-infestation duration on *A. pisum*, we assessed nymphal survival, development, adult longevity, and fecundity using an excised leaf feeding assay [37]. Alfalfa lateral branches pre-infested with *A. pisum* for 24 and 48 h (as described in Section 2.2) were used as treatments, with uninfested branches (0 h) serving as controls. Branches were excised, wrapped with absorbent cotton at the cut ends, and placed individually into 2 mL uncapped centrifuge tubes filled with purified water. Absorbent cotton was placed at the tube opening, and the tubes were then placed in Petri dishes (15 cm diameter, 1.5 cm height) lined with moistened filter paper. One alate adult was introduced into each Petri dish with a fine brush. After 24 h, the alate adult and all but one nymph were removed, leaving a single nymph per dish for subsequent observations. Daily observations were conducted to record nymphal molting, larviposition, and mortality. When leaves showed signs of yellowing or decay, branches were replaced with fresh ones from plants pre-infested for the same duration as the originals. Each aphid was considered an individual replicate, with 60 replicates per treatment. For analysis of nymphal survival rate, the 60 replicates were randomly divided into six groups of 10 individuals each, and the number of surviving nymphs was recorded for each group. All experiments were conducted under the same conditions as those described in Section 2.3.

### 2.5. Metabolite Extraction and UHPLC-Based Mass Spectrometry Analysis of Alfalfa Plants Infested by A. pisum

#### 2.5.1. Untargeted Metabolomics

To characterize the metabolic changes associated with aphid resistance, the 48 h post-infestation time point was selected for untargeted metabolomic analysis based on bioassay results (Section 2.3 and Section 2.4). Pre-infested alfalfa leaves were obtained as described in Section 2.2. Six biological replicates were prepared for each of the following groups: the aphid-infested group (48 hpi; TGN5) and the uninfested control group (0 hpi; CGN5). For each replicate, 100 mg of leaves ground in liquid nitrogen was mixed with ice-cold 80% methanol containing 0.1% formic acid. After incubation on ice for 5 min, the mixture was centrifuged at 15,000× *g* for 5 min at 4 °C. The supernatant was diluted with LC/MS-grade water to a final methanol concentration of 53% and centrifuged again (15,000× *g*, 10 min, 4 °C). The final extract was analyzed using a Vanquish UHPLC system coupled to an Orbitrap Q Exactive™ HF-X mass spectrometer (Thermo Fisher Scientific, Bremen, Germany). Separation was performed on a Hypersil Gold column (100 × 2.1 mm, 1.9 μm) at 40 °C with a flow rate of 0.2 mL/min and a linear gradient over 17 min. The mobile phases consisted of (A) 0.1% formic acid in water and (B) methanol for positive ion mode and (A) 5 mM ammonium acetate (pH 9.0) and (B) methanol for negative mode. Mass spectrometer parameters were as follows: spray voltage 3.2 kV, capillary temperature 320 °C, sheath gas 40 arb, auxiliary gas 10 arb.

Raw data were processed with Compound Discoverer 3.1 (Thermo Fisher Scientific) using a retention time tolerance of 0.2 min, mass tolerance of 5 ppm, signal-to-noise ratio of 3, and minimum intensity of 100,000. QC samples were interspersed throughout the analytical run. Peak areas were corrected using the first QC, and compounds with a coefficient of variation (CV) > 30% across QC samples were removed. Peak intensities were normalized to the total spectral intensity. Metabolites were identified by matching against the mzCloud, mzVault, and NovoMetDB databases, yielding qualitative and relative quantitative data. Additional annotation support was obtained from the KEGG (https://www.genome.jp/kegg/pathway.html, accessed on 20 August 2025), HMDB (https://hmdb.ca/metabolites, accessed on 20 August 2025), and LIPID MAPS (http://www.lipidmaps.org/, accessed on 20 August 2025) databases. Statistical analyses were conducted using R (with the metaX package for PLS-DA). Features with a variable importance in projection (VIP) > 1, *p*-value < 0.05, and |log_2_fold change (FC)| ≥ 1 were considered differentially abundant metabolites. Pathway enrichment analysis was performed on the differential metabolites, with a significance threshold of *p* < 0.05.

#### 2.5.2. Targeted Metabolomics of Flavonoids

Since non-targeted metabolomics revealed flavonoids as the most significantly regulated metabolite class, we further performed a targeted metabolomic analysis for this compound group. For targeted metabolomics, three biological replicates were prepared for each treatment, with 100 mg of leaves pre-infested by pea aphids used per replicate. Sample preparation and UHPLC-MS/MS conditions (including ESI source parameters) were identical to those described in Section 2.5.1, except that the mass spectrometer was operated in multiple reaction monitoring (MRM) mode. Flavonoids were identified by matching retention times and MRM transitions against the NovoMetDB database. Relative quantification was achieved by peak area comparison, and the data were expressed as fold change (FC) between the aphid-infested group (48 hpi; TGN5) and the uninfested control group (0 hpi; CGN5). The criteria for defining differentially abundant metabolites were the same as those described in Section 2.5.1.

### 2.6. Effects of Flavonoids on the Performance of A. pisum

The effects of sakuranetin and chrysin on the survival and fecundity of *A. pisum* were evaluated using a detached *V. faba* leaf bioassay [9,37], because *V. faba* leaves are larger and flatter, remain turgid longer under detached conditions, and allow for more consistent compound delivery and behavioral monitoring. Dimethyl sulfoxide (DMSO), sakuranetin (CAS No.: 2957-21-3; analytical standard, purity ≥ 98% by HPLC), and chrysin (CAS No.: 480-40-0; analytical standard, purity 99.9%) were obtained from Shanghai Yuanye Bio-Technology Co., Ltd. (Shanghai, China). Based on preliminary results and literature reports [9,30,38], each flavonoid was tested at 0.1, 1.0, and 10 μg/μL, with 1% DMSO (*v*/*v*) as the solvent and the same DMSO solution without flavonoids served as the control (0 μg/μL). Excised *V. faba* leaves, with petioles wrapped in absorbent cotton, were inserted into a 1.5 mL centrifuge tube filled with the test solutions. Each tube was placed in a 15 cm Petri dish lined with filter paper pre-moistened with the same test solution. After the solution was fully absorbed by the leaves, ten third-instar nymphs were introduced into each dish. Survival and reproduction of the aphids were recorded every other day over the eight-day observation period, and the test solutions were replenished daily. After the aphids developed into adults, the number of offspring produced was counted, and the newborn nymphs were promptly removed. Survival rate and fecundity (number of nymphs produced per adult) were monitored throughout the experiment, with five biological replicates per treatment.

### 2.7. Statistical Analysis

Data were organized using Excel 2021 and analysed using IBM SPSS Statistics V.19.0. Prior to analysis, percentage data (survival rates and preference proportions) were transformed using an arcsine square-root transformation to improve homogeneity of variance. Normality was assessed using the Shapiro–Wilk test, and homogeneity of variance was assessed using Levene’s test. For fecundity (count data), the normality assumption was not violated (Shapiro–Wilk, *p* > 0.05), permitting parametric tests. Two-way ANOVA was first conducted to evaluate the interactive effects of concentration and treatment time on survival rate and fecundity. Subsequently, one-way ANOVA was performed for each time point separately to compare concentration differences, followed by Tukey’s HSD post hoc test for multiple comparisons (*p* < 0.05). Feeding preference data of apterous adults toward aphid-infested alfalfa branches were analyzed using a paired *t*-test. For the targeted metabolomics analysis, independent-samples *t*-tests were used to compare the relative contents of sakuranetin and chrysin between pre-infestation and post-infestation samples. A *p*-value < 0.05 was considered statistically significant. All figures were generated using GraphPad Prism V.10.0 (GraphPad Software, Inc., San Diego, CA, USA).

## 3. Results

### 3.1. Preference of A. pisum to Conspecific-Infested Alfalfa Plants

**Figure 1:**
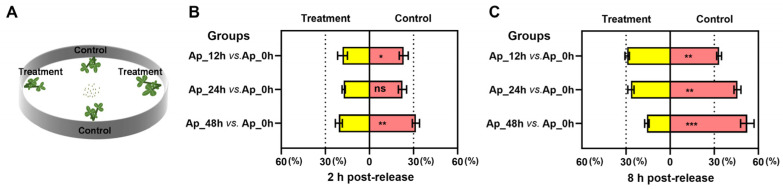
Feeding preference (%) of *A. pisum* apterous adults to conspecific-infested alfalfa branches. (**A**) Schematic diagram of the choice-test experimental design. (**B**) Preference at 2 h post-release. (**C**) Preference at 8 h post-release. Each dish contained one uninfested control branch (0 h) and one branch pre-infested by conspecifics for 12, 24, or 48 h. The proportion (%) of aphids settled on each branch was calculated as (number of aphids on test branch)/(total number of settled aphids) × 100%. Data are presented as mean percentage ± SE. Asterisks beside the error bars indicate significant differences between the two infestation durations within each comparison group (paired *t*-test, n = 6 per comparison group); * *p* < 0.05, ** *p* < 0.01, *** *p* < 0.001; “ns” denotes no significant difference.

The feeding preference of apterous adult *A. pisum* for alfalfa lateral branches pre-infested by conspecifics for 12, 24, or 48 h (relative to uninfested controls, 0 h) is shown schematically (Figure 1A) and quantitatively (Figure 1B,C). At 2 h post-release, no significant preference difference was observed between the 24 h pre-infested plants and the controls (Ap_24h *vs*. Ap_0h: t = 1.732, df = 5, *p* = 0.144). In contrast, adults significantly avoided plants pre-infested for 12 h (Ap_12h *vs*. Ap_0h: t = 3.873, df = 5, *p* = 0.012) and 48 h (Ap_48h *vs*. Ap_0h: t = 4.540, df = 5, *p* = 0.006) (Figure 1B). At 8 h post-release, adults significantly preferred uninfested (control) lateral branches over pre-infested ones for all pre-infestation durations tested (Ap_12h *vs*. Ap_0h: t = 5.000, df = 5, *p* = 0.004; Ap_24h *vs*. Ap_0h: t = 5.861, df = 5, *p* = 0.002; Ap_48h *vs*. Ap_0h: t = 8.451, df = 5, *p* < 0.001) (Figure 1C).

### 3.2. Performance of A. pisum on Conspecific-Infested Alfalfa Plants

**Figure 2:**
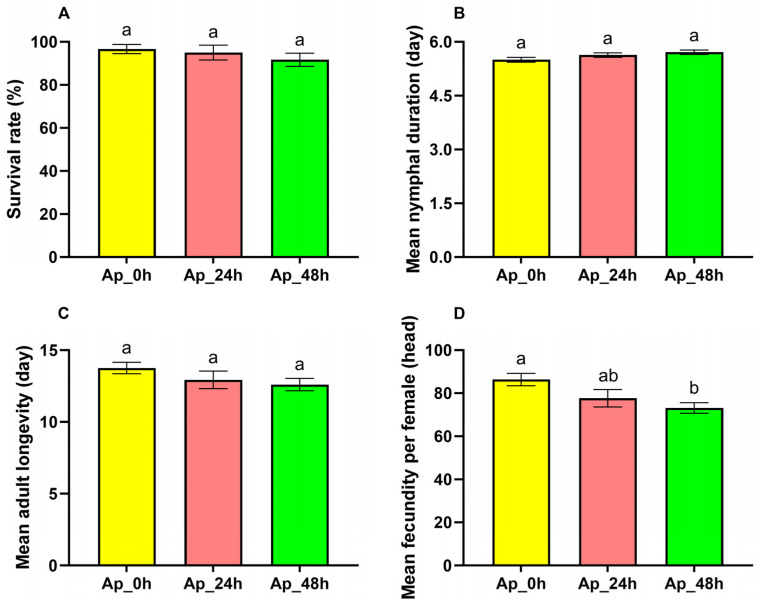
Survival, development, longevity, and reproduction of *A. pisum* on conspecific-pre-infested alfalfa plants. (**A**) Nymphal survival rate (%) = (number of surviving nymphs at end of observation/Initial number of nymphs) × 100. (**B**) Nymphal duration (days) = (date of adult eclosion − date of birth (day 0)). (**C**) Adult longevity (days) = (date of death − date of adult eclosion). (**D**) Fecundity = total number of nymphs produced over adult lifespan/Number of females. For survival analysis, 60 nymphs per treatment were divided into six groups of 10 individuals (n = 6 biological replicates). For development time, longevity, and fecundity, individual aphids were followed (n = 60 per treatment). Data are presented as mean ± SE. Different letters above the error bars indicate significant differences among treatments (one-way ANOVA followed by Tukey’s HSD post hoc test, *p* < 0.05); treatments sharing the same letter denote no significant difference.

The key biological parameters of *A. pisum* fed on alfalfa plants pre-infested by conspecifics for different durations are shown in Figure 2. No significant differences were observed among treatments in nymphal survival rate (*F* = 0.813, df = 2, 15, *p* = 0.462, Figure 2A), nymphal duration (*F* = 2.707, df = 2, 167, *p* = 0.070, Figure 2B), or adult longevity (*F* = 1.489, df = 2, 167, *p* = 0.228, Figure 2C), whereas mean fecundity per female differed significantly among treatments (*F* = 4.375, df = 2, 167, *p* = 0.014, Figure 2D). Specifically, survival rate exhibited a decreasing trend with prolonged pre-infestation duration, declining from 96.67% (Ap_0h) to 91.67% (Ap_48h) (Figure 2A). In contrast, nymphal duration increased from 5.50 days (Ap_0h) to 5.71 days (Ap_48h) (Figure 2B), and adult longevity gradually shortened from 13.76 days (Ap_0h) to 12.60 days (Ap_48h) (Figure 2C). Although mean fecundity per female did not differ significantly between the control (Ap_0h) and Ap_24h treatments (*p* = 0.133), it was significantly lower in the Ap_48h treatment than in the Ap_0h treatment (*p* = 0.012) (Figure 2D).

### 3.3. Untargeted Metabolomic Analysis of Alfalfa Leaves in Response to A. pisum Infestation

**Figure 3:**
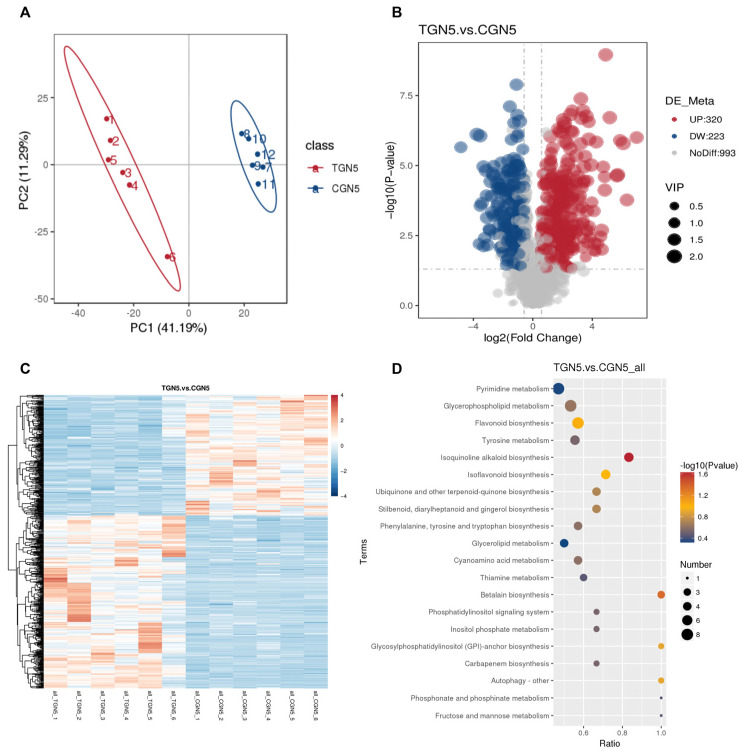
Analysis of all accumulated metabolites in alfalfa under *A. pisum* infestation. (**A**) PCA scores plot showing separation between control (uninfested, blue) and 48 h post-infestation (red) samples (n = 6 biological replicates per group). (**B**) Bubble plot of upregulated (red) and downregulated (blue) metabolites. (**C**) Heatmap of all detected metabolites. (**D**) KEGG enrichment analysis of all detected metabolites.

To identify potential defensive metabolites against the pea aphid in the alfalfa variety GN5, we performed non-targeted metabolomic analysis on control plants and plants infested with aphids for 48 h. Principal component analysis (PCA) revealed a distinct separation between the two treatment groups, with PC1 and PC2 accounting for 41.19% and 11.29% of the total variance, respectively (Figure 3A). An overview of all accumulated metabolites is presented in Figure 3B,C. Compared with the uninfested controls, a total of 543 DAMs were identified following aphid feeding, of which 320 were upregulated and 223 were downregulated (Table S1). KEGG enrichment analysis of the DAMs highlighted several key plant defense-related metabolic pathways, most notably the flavonoid and isoflavonoid biosynthesis pathways (Figure 3D), indicating that flavonoids and isoflavonoids are important classes in response to pea aphid feeding. In these two pathways, 45 and 13 related metabolites were identified, respectively (Table S2). Specifically, within the flavonoid biosynthesis pathway, 18 metabolites were significantly upregulated, while only 4 were downregulated. Remarkably, all eight identified metabolites in the isoflavonoid biosynthesis pathway were upregulated (Table S2).

### 3.4. Targeted Flavonoid Metabolomic Analysis of Alfalfa Leaves in Response to Aphid Infestation

**Figure 4:**
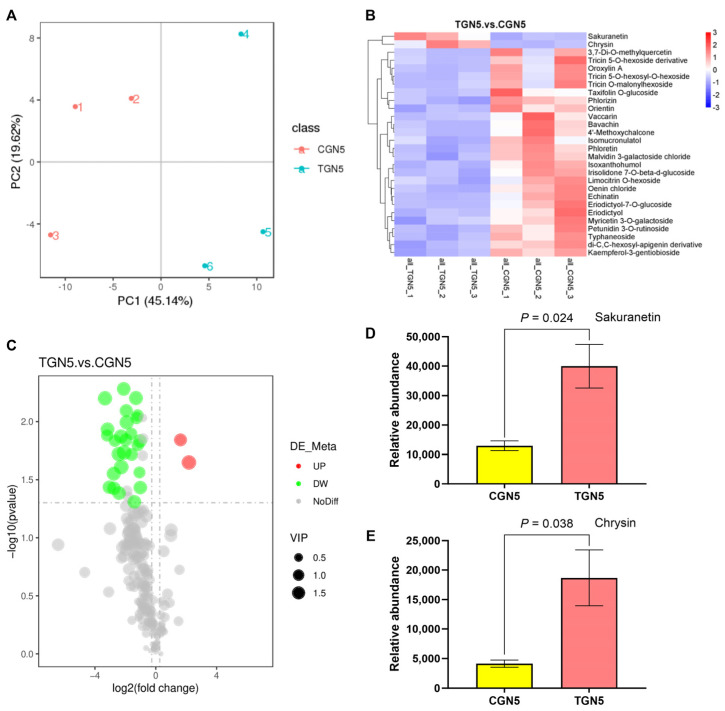
Analysis of differential flavonoids in alfalfa under *A. pisum* infestation. (**A**) PCA score plot showing separation between control and 48 hpi samples. (**B**) Heatmap of 28 identified flavonoids. (**C**) Bubble plot of upregulated (red) and downregulated (green) flavonoids. Relative abundance of sakuranetin (**D**) or chrysin (**E**) in control *vs*. 48 hpi samples. Data are presented as mean peak area ± SE (n = 3). Student’s *t*-test was used for comparisons between groups.

To compare flavonoid composition in GN5 alfalfa leaves before and after aphid feeding, a targeted metabolomic analysis was further conducted. PCA revealed a clear separation between the aphid-infested and control samples along the first principal component, indicating that a significant alteration in the flavonoid profile was induced by aphid feeding (Figure 4A). Using heatmap and bubble plot analyses, a total of 28 flavonoids were identified (Figure 4B,C). Among these 28 flavonoids, 26 were significantly downregulated, whereas only two—sakuranetin and chrysin—were significantly upregulated (fold change > 2, *p* < 0.05) (Table S3). Notably, after 48 h of pea aphid feeding, the relative abundances of both flavonoids increased markedly, with fold changes of 3.09 and 4.53, respectively (Figure 4C,D).

### 3.5. Effects of Sakuranetin and Chrysin Mediated by Faba Bean Leaves on A. pisum

The biological activities of two key flavonoids, chrysin and sakuranetin, against the pea aphid were assessed. Two-way ANOVA revealed significant interactions between concentration and time for all parameters examined, except for the effect of chrysin on aphid mortality, which was non-significant (Table 1).

**Figure 5:**
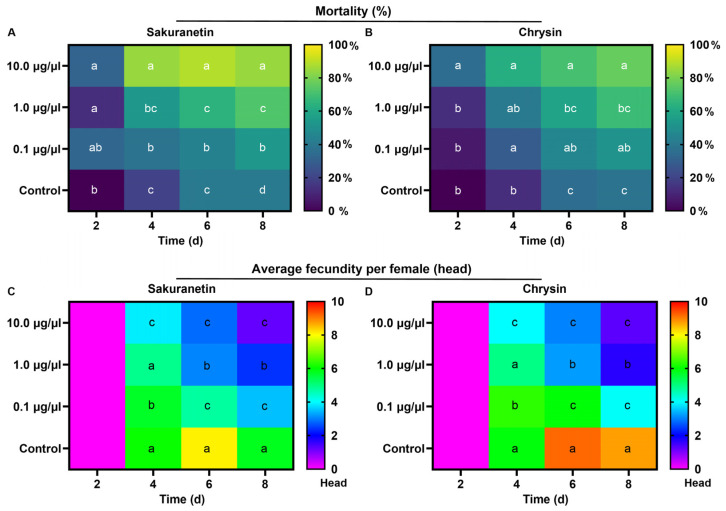
Effects of sakuranetin and chrysin on mortality and fecundity of *A. pisum*. Mortality rate (%) at 2, 4, 6, and 8 days of aphid exposure to sakuranetin (**A**) or chrysin (**B**) at concentrations of 0 (control, 1% DMSO), 0.1, 1.0, or 10.0 μg/μL. Fecundity (number of nymphs produced per female) at 2, 4, 6, and 8 days of exposure to sakuranetin (**C**) or chrysin (**D**) at the same concentrations. Data are presented as mean ± SE (n = 5 biological replicates, with 10 aphids per replicate). Tukey’s HSD post hoc test was used for multiple comparisons. Different letters indicate significant differences between concentrations at the same time point (*p* < 0.05); comparisons were not made across different time points.

For sakuranetin, at 2 days post-treatment, the 10.0 and 1.0 μg/μL groups had significantly higher mortality than the control (*F* = 7.603, df = 3, 16, *p* = 0.002), reaching 31.42% and 12.87%, respectively. At 4, 6, and 8 days post-treatment, the mortality rate in the 10.0 μg/μL group was significantly higher than that in all other treatment groups (4 days: *F* = 16.215, df = 3, 16, *p* < 0.001; 6 days: *F* = 39.100, df = 3, 16, *p* < 0.001; 8 days: *F* = 45.263, df = 3, 16, *p* < 0.001) (Figure 5A). For chrysin, at 2 days post-treatment, the mortality rate in the 10.0 μg/μL group was significantly higher than that in the other treatment groups (*F* = 8.075, df = 3, 16, *p* = 0.0017), reaching 35.01%. At 4 days post-treatment, the 10.0 and 0.1 μg/μL groups had significantly higher mortality rates than the control (*F* = 7.327, df = 3, 16, *p* = 0.0026). At 6 and 8 days post-treatment, mortality was lowest in the control and highest in the 10.0 μg/μL group (6 days: *F* = 13.699, df = 3, 16, *p* < 0.001; 8 days: *F* = 13.752, df = 3, 16, *p* < 0.001), reaching 70.40% and 76.53%, respectively (Figure 5B). For fecundity, at 4 days post-treatment, the highest values were observed in the control and 1.0 μg/μL groups for both flavonoids (sakuranetin: 6.1 and 5.7 nymphs per female, respectively; chrysin: 5.9 and 6.3 nymphs per female, respectively), whereas the lowest values were in the 10.0 μg/μL group (sakuranetin: *F* = 30.905, df= 3, 16, *p* < 0.001; chrysin: *F* = 40.919, df = 3, 16, *p* < 0.001). At 6 and 8 days post-treatment, the lowest fecundity was observed in the 10.0 and 0.1 μg/μL groups for both compounds, significantly lower than that in the control group (6 days: sakuranetin, *F* = 325.603, df = 3, 16, *p* < 0.001; chrysin, *F* = 530.412, df = 3, 16, *p* < 0.001; 8 days: sakuranetin, *F* = 89.874, df = 3, 16, *p* < 0.001; chrysin, *F* = 421.733, df = 3, 16, *p* < 0.001) (Figure 5C,D). Therefore, for sakuranetin, a concentration of 0.1 μg/μL was sufficient to significantly inhibit aphid reproduction, whereas 10.0 μg/μL was required to significantly reduce survival. For chrysin, both survival and reproduction were significantly reduced at 0.1 μg/μL.

## 4. Discussion

When attacked by herbivores, plants produce toxic secondary metabolites, including alkaloids, phenolic acids, and flavonoids, which exhibit antifeedant properties or interfere with herbivore growth and development [9,39,40,41,42]. In recent years, the application of these feeding deterrents and growth inhibitors in pest management has garnered considerable attention, as they mediate plant–insect interactions and profoundly influence insect behavior [9,30,43]. In the present study, behavioral and biological assays revealed that *A. pisum* displayed marked avoidance responses toward conspecific pre-infested alfalfa leaves, and prolonged pre-infestation significantly reduced aphid reproductive capacity. Metabolomic analysis further demonstrated that aphid feeding induced metabolic reprogramming of the flavonoid and isoflavonoid biosynthesis pathways in GN5, with sakuranetin and chrysin showing specific accumulation. Both compounds inhibited *A. pisum* fecundity at different concentrations. Notably, chrysin appeared to reduce aphid survival even at a relatively low concentration, suggesting a potentially stronger toxic effect.

Plants possess both constitutive and induced defense mechanisms. Compared with constitutive resistance, induced resistance is more energy efficient and flexible, enabling rapid activation upon herbivore attack and exerting significant effects on subsequent feeding by the same or different insect species. Our results further showed that pea aphids exhibited significant avoidance of plants pre-infested for 12 h and 48 h (but not 24 h) as early as 2 h post-release. By 8 h post-release, they significantly avoided all pre-infested plants. Furthermore, prolonged pre-infestation lasting 48 h significantly reduced mean fecundity per female, whereas no significant differences were observed in nymphal survival, developmental duration, or adult longevity. These findings indicate that the fitness cost of prolonged infestation is manifested primarily in reproduction rather than survival. Similar phenomena have been documented in other plant-herbivore systems. For instance, the whitefly *Bemisia tabaci* MEAM1 significantly avoids conspecifics-infested plants, a response associated with the induced biochemical changes in cabbage, including increased phenolics, flavonoids, and soluble protein, as well as upregulated expression of phenylpropanoid pathway genes (*PAL2*, *C4H*, and *4CL*) [40]. In contrast, research on the brown planthopper–rice system revealed a different strategy: nymphal feeding suppressed plant volatiles induced by gravid females that normally attract natural enemies, thereby reducing parasitism rates [44]. Based on these findings, we propose that herbivorous insects may employ divergent strategies to cope with plant-induced defenses. Pea aphids appear to exploit the temporal lag in defense activation to circumvent reproductive costs, as their avoidance behavior is time-dependent. The brown planthopper reduces parasitism risk through transgenerational signal interference, facilitating population development [44]. MEAM1 whiteflies, in turn, avoid conspecific-infested plants in response to phenolic and flavonoid accumulation [40]. These distinct strategies collectively illustrate the sophisticated responses of insects to plant defenses and the underlying plant–insect coevolutionary dynamics. Furthermore, the avoidance and reduced performance of pea aphids on conspecific-pre-infested leaves may be attributable to the accumulation of flavonoids at the feeding sites induced by prior infestation. In the high-flavonoid maize line *ZmY1*, herbivory by *Helicoverpa zea* induced local flavonol accumulation around feeding sites, resulting in elevated local concentrations of flavonoids—particularly 3-deoxyanthocyanidins and flavan-4-ols—which directly damaged the larval midgut, induced leaky-gut syndrome, and increased mortality [43]. Finally, this avoidance behavior may also be closely associated with the ability of flavonoids to interfere with aphid feeding behavior [45]. Nevertheless, the specific mechanism underlying the avoidance response of pea aphids toward conspecific-pre-infested GN5 alfalfa plants remains to be further elucidated.

Non-targeted metabolomic analysis revealed that differential metabolites in alfalfa leaves were significantly enriched in the flavonoid biosynthesis pathway at 48 h following aphid infestation, indicating the involvement of flavonoid compounds in the defense response against the pea aphid. This finding is consistent with the results reported by Wang et al. [46], in which the phenylpropanoid and flavonoid metabolism played important roles in wheat kernel resistance to the wheat blossom midges (*Sitodiplosis mosellana*). Furthermore, in thrips-resistant alfalfa varieties, defense-related genes and proteins were induced through the regulation of genes involved in the salicylic acid and flavonoid biosynthesis pathways, thereby enhancing plant defense capabilities [27]. Collectively, these findings demonstrate that the flavonoid biosynthesis pathway in legumes plays a critical role in the resistance of insect-resistant varieties against pest infestation.

Targeted metabolomic analysis was subsequently performed to measure the relative abundance of flavonoid compounds in alfalfa leaves following aphid feeding, based on peak comparisons across sample groups. Notably, only two compounds, sakuranetin and chrysin, showed significant increases in relative abundance, with fold changes of 3.09 and 4.53, respectively. Similarly, in the resistant peanut cultivar TF22, chrysin levels were significantly enriched in leaves following feeding by *F. occidentalis*, with a twofold increase compared to pre-infestation levels [46]. Accordingly, sakuranetin and chrysin are suggested to be candidate secondary metabolites that may help protect alfalfa from further infestation by the pea aphid. Nevertheless, their specific roles in GN5 resistance remain to be determined.

Extensive research has been conducted on the insecticidal activity of flavonoids [9,14,30,47]. For instance, seven flavonoids, including kaempferol and naringenin, significantly inhibited the settling of *A. gossypii* on cotton plants, reduced honeydew secretion, shortened adult longevity, and decreased fecundity [9]. Similarly, larvae of the corn earworm (*H*. *zea*) fed on high-flavonoid maize lines showed elevated mortality and reduced body weight [43]. In another study, feeding by the pod borer (*Helicoverpa armigera*) on *Cajanus platycarpus* induced significant upregulation of chalcone synthase (*CHS*), flavonoid-3′, 5′-hydroxylase (*F3*′*5*′*H*), and flavonol synthase (*FLS*) genes, accompanied by significant accumulation of naringenin, kaempferol, and quercetin. When these flavonoids were added to the artificial diet of the pod borer, larval duration was prolonged [48]. Sakuranetin, a flavonoid phytoalexin with known antimicrobial and antifungal activities, was first identified as the aglycone of sakuranin from cherry tree bark in 1908 [49]. Its production has also been demonstrated in rice following UV irradiation or *Magnaporthe oryzae* infection, where it functions as a phytoalexin active against multiple fungal pathogens [38,50,51]. Recent studies have further indicated that sakuranetin can act as an insect resistance signaling molecule in host plants. For example, Liu et al. [38] demonstrated that *N. lugens* infestation induced sakuranetin accumulation in rice phloem sap to an estimated concentration of 0.5 μg/mL, which significantly reduced insect survival in artificial diet assays. Consistent with this, the present study showed that sakuranetin at 0.1 μg/μL significantly inhibited pea aphid reproduction, while 10.0 μg/μL significantly reduced their survival.

The insecticidal activity of chrysin has been further validated in other insect species. For example, dietary chrysin (5–3125 ppm) prolonged larval development, reduced pupation and adult emergence rates, and decreased larval weight and growth rate in the melon fruit fly (*Zeugodacus cucurbitae*). Chrysin also exhibited oviposition deterrent activity under both choice and no-choice conditions [52], paralleling our observation that chrysin at 0.1 μg/μL reduced survival and fecundity of pea aphids. Regarding its mode of action, chrysin is thought to affect the digestive or nervous systems of insects. Supporting this, chrysin isolated from *Acorus calamus* showed potent activity against *Spodoptera litura* larvae (LD_50_ = 2.752 µg per larva), outperforming the crude extract [53]. Furthermore, chrysin treatment reduced carboxylesterase activity in treated larvae, suggesting that inhibition of detoxification enzymes may contribute to its insecticidal mechanism [53].

Nevertheless, future studies need to determine whether the accumulation of sakuranetin and chrysin in GN5 induced by pea aphid feeding represents a general response to aphid infestation, a race-specific accelerated defense unique to this resistant genotype, or a causal mechanism of aphid resistance [54]. This distinction is important because susceptible hosts can sometimes exhibit metabolic responses to or even greater than those observed in resistant genotypes. The mode of action of these two flavonoids—whether they alter aphid feeding behavior, suppress detoxification enzyme genes, or are merely associated with broader defense responses—requires further elucidation using exogenous supplementation, genetic manipulation, or near-isogenic lines with contrasting flavonoid profiles. Furthermore, although *V. faba* is a suitable host for *A. pisum* [9], it is not the target crop, and due to differences in leaf anatomy, compound stability, and translocation efficiency, the absolute concentrations of sakuranetin and chrysin reaching the phloem sap may differ between *V. faba* and alfalfa. Therefore, the actual contribution of these two compounds to GN5 resistance in alfalfa still needs to be validated using alfalfa leaves or whole plants in future studies.

## 5. Conclusions

In summary, this study demonstrates that infestation by the pea aphid induces a strong defense response in the resistant alfalfa variety GN5, which significantly affects the host preference and performance of the aphid. Non-targeted and targeted metabolomic analyses revealed that the flavonoids sakuranetin and chrysin accumulate significantly following aphid feeding and are enriched in the flavonoid biosynthesis pathway. Exogenous feeding assays further confirmed that elevated concentrations of these two compounds increase aphid mortality and reduce fecundity, supporting their identification as candidate bioactive compounds contributing to GN5 defense. Notably, chrysin exhibits stronger insecticidal activity than sakuranetin, effectively reducing both aphid survival and reproduction at a relatively low concentration of 0.1 μg/μL, whereas sakuranetin requires a 100-fold higher concentration to achieve a comparable reduction in survival. Overall, these findings reveal the chemical basis of resistance in GN5 and highlight chrysin and sakuranetin as promising lead compounds for future biopesticides. Future studies are required to elucidate the underlying molecular mechanisms, including jasmonate-mediated regulation, and—most importantly—to conduct field-based evaluations to ultimately assess their practical applicability for advancing sustainable forage.

## Figures and Tables

**Table 1:** Two-way ANOVA analysis of concentration and time effects on *A. pisum* mortality and fecundity.

Biological Parameters	Source of Variation *	*df*	*F*	*p*
Sakuranetin-Mortality	C	3	81.737	<0.0001
	T	3	83.160	<0.0001
	C × T	9	5.968	<0.0001
	Error	64		
Sakuranetin-Aphid fecundity	C	3	300.454	<0.0001
	T	3	946.394	<0.0001
	C × T	9	55.910	<0.0001
	Error	64		
Chrysin-Mortality	C	3	44.357	<0.0001
	T	3	62.254	<0.0001
	C × T	9	0.639	0.759
	Error	64		
Chrysin-Aphid fecundity	C	3	714.820	<0.0001
	T	3	1440.543	<0.0001
	C × T	9	164.030	<0.0001
	Error	64		

* Two-way ANOVA was conducted to evaluate the interactive effects of compound concentration and treatment time on the survival rate and fecundity of pea aphids. Tukey’s HSD post hoc test was performed for multiple comparisons to determine the significance of differences among different concentrations at the same time point (*p* < 0.05). C = Concentration, T = Time.

## Data Availability

Data can be provided upon request to the corresponding author or the first author.

## Supplementary Materials

The following supporting information can be downloaded at: https://www.mdpi.com/article/10.3390/insects17070679/s1, Table S1: Overview of differential metabolites identified in alfalfa leaves in response to pea aphid (*Acyrthosiphon pisum*) feeding. Table S2: Overview of differential flavonoid metabolites identified in GN5 alfalfa leaves in response to pea aphid feeding using untargeted metabolomics. Table S3: Overview of all differential metabolites identified in GN5 alfalfa leaves in response to pea aphid feeding using targeted metabolomics.
